# Valid and reliable neonatal near-miss assessment scale in Ethiopia: a psychometric validation

**DOI:** 10.1080/16549716.2022.2029334

**Published:** 2022-02-02

**Authors:** Mengstu Melkamu Asaye, Kassahun Alemu Gelaye, Yohannes Hailu Matebe, Helena Lindgren, Kerstin Erlandsson

**Affiliations:** a Department of Women and Family Health, School of Midwifery, College of Medicine and Health Sciences, University of Gondar, Gondar, Ethiopia; b Department of Epidemiology and Biostatistics, Institute of Public Health, College of Medicine and Health Sciences, University of Gondar, Gondar, Ethiopia; c Department of Pediatrics and Child Health, School of Medicine, College of Medicine and Health Sciences, University of Gondar, Gondar, Ethiopia; d Department of Women’s and Children’s Health, Karolinska Institute, Solna, Sweden; e Institution for Health and Welfare, Dalarna University, Falun, Sweden

**Keywords:** Construct validity, composite reliability, average variance extracted, factor analysis, neonatal near miss scale

## Abstract

**Background:**

The concept of a neonatal near miss is used to explain neonates who nearly died but survived a life-threatening complication in the first 28 days of life. We have left many ill surviving (near-miss) neonates, due to a lack of valid and reliable assessment scale, particularly in Ethiopia.

**Aim:**

We aim to psychometrically validate the neonatal near-miss assessment scale (NNMAS) for Ethiopia.

**Methods:**

A total of 465 live birth neonates were included with the assumption of a participant-to-item ratio of 15:1. A new contextually validated NNMAS was used to collect data. The Kaiseri––Mayer––Olkin (KMO) measure of sampling adequacy with a cutoff value of ≥0.50 for each item was applied. For reliability and validity of NNMAS, exploratory factor analysis using principal component analysis with oblique varimax rotation was used. Internal consistency and reliability were assessed using Cronbach’s alpha. Convergent and discriminant validity was assessed using composite reliability (CR) and average variance extracted (AVE).

**Results:**

The Kaiser––Mayer––Olkin (KMO = 0.74) measure of sampling adequacy and Bartlett’s Sphericity test for the appropriateness of the identity matrix (*χ*^2^ **= **2903.9, df = 276, and P = 0.000) were suitable for exploratory factor analysis (EFA). The correlation matrix determinant of the study was 0.002. The principal component analysis (PCA) identified six factors and together explained 54.3% of the variation in the Neonatal Near miss. The Cronbach-alpha coefficient was 0.80 for the entire scale. The composite reliability values of the factors ranged from 0.87 to 0.95. The AVEs, CR, and factor loadings were above 0.5 for all factors indicating that convergent validity was met. The square roots of the AVEs were greater than factor correlation values. It was revealed that discriminated validity was also met.

**Conclusion:**

The neonatal near-miss assessment scale was found to be valid and reliable in the present context. The scale can be used to identify near-miss neonates in Ethiopia.

## Background

Neonatal mortality is expected to be reduced to at least as low as 12 deaths per 1,000 live births, according to the Global Sustainable Development Goals (SDGs) agenda seeking to be achieved by 2030. The proposed SDG target for neonatal mortality is to prevent the deaths of newborns [1]. Ethiopia planned to reduce the neonatal mortality rate (NMR) from 29 in 2015/16 to 11 per 1,000 live births by 2019/2020 [2], but the NMR increased to 30 per 1,000 live births in 2019 [3]. The highest mortality rate was in the Amhara Regional State accounted about 47 per 1,000 live births [4].

Neonatal mortality is a significant public health problem in many low-resource countries [5] yet for every death, there are more than eight newborns that suffer life-threatening complications but survive (near-miss) [6]. The near-miss concept is being recommended in the neonatal context to accelerate progress towards achieving SDGs [7]. Emphasis on the newly emerging concept could help to identify the quality of care issue and strengthen clinical practices to prevent neonatal deaths [8].

The concept of NNM is used to identify neonates who nearly died but survived from a life-threatening complication in the first 28 days of life [9,10]. It is survival for the first 7 days of life or before discharge from life-threatening complications [7,11]. The conceptualization and validation of a neonatal near-miss scale in local contexts needs attention for further development of the near-miss concept and identification of neonates who nearly died but survived for quality of care audit, identification of care issues, and strengthening of clinical practice [12].

Several studies have presented scoring tools used to assess severe neonatal morbidities, but none of these scoring markers can be used to define near-miss neonates [11–13]. The emerging pragmatic criteria are birth weight under 1750 g, APGAR scores under 7 at 5 minutes and gestational age under 33 completed gestation weeks [7,14–18]. The management criteria are phototherapy within 24 hours of life, cardiopulmonary resuscitation, use of vaso-active drugs, anticonvulsants, blood product or surfactant utilization, surgery, or use of steroids for treatment of refractory hypoglycemia, or intubation for 7 days [10,19–21] and one study used certain clinical criteria [22]. Lab-investigation criteria were not included but it could be feasible in low resource countries like Ethiopia. The validated neonatal near-miss assessment scale should be simple to use and easy to understand [7].

The absence of a clear and consistent conceptualization of neonatal near-miss assessment-scale appraisal limits the ability to inform health-care providers, policymakers, and clinical practices [23]. This challenges the reduction of neonatal mortality [6,7,12,14]. In the local context, the developed neonatal near-miss criteria could be used for the measurement of quality of neonatal care and the evaluation of death reviews [24,25]. We recently published the face and content validated NNMAS [26]; however, there is still a need for psychometric validation and reliability checks as well.

Thus, a broad understanding of vulnerable and severely life-threatening events for neonates and a validated NNMAS can assist health-care providers and policymakers in overcoming barriers to quality-of-care provision in low resource-limited countries such as Ethiopia. This makes the psychometrically validated NNMAS could improve the reduction of neonatal mortality in Ethiopia [6,8,9,13,24,25]. Therefore, in this study, we set out to psychometrically validate NNMAS for Ethiopia. We hypothesize that the neonatal near-miss assessment scale is reliable and valid.

## Methods

### Domain and item generation

We conducted a literature review, expert panel discussions, and content analysis for neonatal near-miss case identification in item and domain development. The panel of experts was selected considering expert knowledge, specific training, or professional experience on the subject matter. We gained valuable feedback from panel experts. Nine expert panel members were involved in the first phase of judgments. These experts confirmed that the scale is ready for quantification through the meeting. To minimize over- or under-estimation of the quantifications, ten other independent panels of experts were invited for the second round to rate the necessity, relevancy, and clarity of each selected item in measuring the related domains. Then, four domains with 31 items were approved for the identification of near-miss neonates in content validation and published [26].

### Study setting and population

An institution-based cross-sectional design was employed. This study was conducted in four randomly selected hospitals (University of Gondar comprehensive specialized hospital, Dembia primary hospital, Addis Zemen primary hospital, and Felegehiwot comprehensive specialized hospital) at Maternity and Neonatal Wards in Amhara region, between December 2020 and March 2021. Each maternity ward has triage, follow-up, second stage, and postnatal units. The neonatal ward also has different partitions. Senior Doctors, Residents, Midwives, and Nurses were working in each ward. During the study period, the average number of births ranged from 120 to 340 births per month.

Annually, of 13,640 deliveries at maternity wards, 2340 neonates were admitted to the neonatal wards where data was collected on live birth neonates after permission from their parents. None of the newborns’ mothers refused to let their neonates participate in the study. The inclusion criteria included singletons and live birth neonates who were delivered at each hospital. Twin, stillbirth, and readmitted neonates were excluded from the study.

### Sample

The sample size determination for the psychometric analysis was performed with an assumption [27] of subject-to-item ratio (15:1). A total of 465 live birth neonates were involved in this study validating the items in NNMAS. The study participants were selected using a systematic random sampling method from live birth neonates who were delivered to the selected hospitals.

### Procedures

Trained BSc midwives were recruited for data collection. Patients were identified through patient lists generated by data collectors at the wards. Written consent was obtained from the neonate’s mother before data collection began. Mothers of neonates were given written and oral information on the aims and procedures of the study. It was stressed that participation was voluntary and that non-participation would not affect the care and treatment of neonate. Informed consent was obtained in writing or by thumbprint. The neonates were observed by the data collectors and data gathered from the records of the neonate and the mother. Additional demographic and clinical data were collected from the mothers and the records at the ward. The chart review questionnaires took 20 minutes and were performed at the neonatal wards.

### Description of NNMAS

A content-validated neonatal near-miss assessment scale with 31 items was employed. The developed and validated scale to assess neonatal near-miss cases among live birth neonates is called the Neonatal Near-miss Assessment scale. The scale has 24 items, which are categorized into dichotomous variables (yes/no) [26].

### Psychometric validation of NNMAS

#### Statistical analysis

Inter-item correlations examine the extent to which scores on one item are related to scores on other items in a score. An inter-item correlation of ≥0.30 in absolute value for each item was considered desirable to conduct a factor analysis [28]. Item-total correlations were also carried out to assess the extent to which an item was correlated to the overall scale. The Pearson correlation coefficient values of the corrected item-total correlation ≥0.20 [29] were considered satisfactory and included in the scale.

The data were entered using Epi-info version 7 and analyzed using Statistical Package for Social Science (SPSS) version 24.

We used exploratory factor analysis (EFA), which is a statistical technique, to identify the smallest number of factors that can be explained by numerous observed variables [30] and to group items into a set of easily interpretable factors. Preliminary analysis regarding inter-item correlation, KMO measure of sampling adequacy, and Bartlett’s Test of Sphericity was carried out to determine the factorability of the scale [27]. The KMO measure of sampling adequacy with the recommended value ≥of 0.70 for the overall items was considered for the appropriateness of the data for EFA [28]. A significant value of less than 0.05 indicates that the data do not produce an identity matrix and are acceptable for further analysis [31].

Principal Component Analysis (PCA) method was used for data extraction. The communalities that were explained by the combination of extracted items were set at a minimum value of 0.3 to retain an item. A factor with Eigen values ≥1 was used to determine the optimal number of underlying factors to be extracted. The factor loading value was set at≥0.30 and considered appropriate to retain an item in each factor [32]. Factors obtained during factor extraction were rotated using a varimax rotation procedure with Kaiser Normalization. The inter-factor correlation and the strength of correlation between factors were checked by observing the component correlation matrix. The inter-factors correlation > 0.30 in absolute value was considered by choosing the oblique rotation method.

#### Reliability

Cronbach’s alpha and composite reliability were used to determine the internal consistency reliability. The statistical value greater than or equal to 0.70 was accepted as evidence of good internal consistency for the scale [33]. In the current study, 0.25 was taken as a lower limit for the item-total correlation [34].

#### Content validation

Content validity ratio, content validity index (item and scale levels), and kappa coefficient of the agreement were used to evaluate the necessity and relevance of the items. The overall S-CVI (universal) for the 31-item scale was from 0.78 to 1, and the overall S-CVI (average) of NNMAS was found to be 0.96 two months before the current study [26].

#### Construct validity

The measurement techniques have to construct validity if it is related to things to which we expect the concept we are trying to be related and independent of those things of which the concept should be independent (convergent and discriminant validity), respectively [23,35,36]. In the current study, principal component analysis and Varimax rotation were used from exploratory factor analysis methods to explore the factor structure of the Neonatal Near-miss Assessment scale.

#### Convergent validity

It is a method to test construct validity [23]. Convergent validity was assessed by factor loading, Composite Reliability (CR), and Average Variance Extracted (AVE) [37]. Exploratory factor analysis was conducted to determine the number of factor retention and factor loadings of variables. All factors had at least three items. The acceptable factor loading value was greater than 0.5. The level of composite reliability is another guideline to review the convergent validity, and the acceptable value of CR is 0.7 and above [38]. Average variance extraction (AVE) measures the level of variance captured by a construct versus the level due to measurement error and its value of more than 0.7 is considered very [38].

#### Discriminant validity

This is a test to ensure that there is no significant variance among different variables that could have the same reason. Discriminant validity is assessed by comparing the square root of AVE with correlations between factors. In order to accept the discriminant validity, the level of the square root of AVE should be greater than the correlations involved in the factors [37].

## Results

### Neonatal medical characteristics

Among 465 live-born neonates, 248 (53.3%) of them were males. Three-quarters of the neonates were under-wrapped and maternal arms in the first 2 hours. This study revealed that 132 (28.4%) of neonates were admitted to the neonatal intensive care unit (NICU). The main reason for admission was the difficulty of breathing shared 55 (41.7%). The ways of transfer to NICU were 87.8% through staff and 12.2% maternal arms (Table 1).

**Table 1.** Characteristics of study neonates in Amhara National Regional State Health Bureau Public Hospitals Northwest Ethiopia 2020 (n = 465)

Variables	Category	Number (%)
Sex of the neonate	Male	248 (53.3)
	Female	217 (46.7)
Care during the first 2 hrs	Wrapped and in maternal arms	352 (75.7)
	Warmer	71 (15.2)
	Others*	42 (9.1)
NICU admission	Yes	132(28.4)
	No	333(76.6
Reason of NICU admission	Difficulty of breathing	55(41.7)
	Anemia	22(16.7)
	Infection	17(12.9)
	Low birth weight	16(12.1)
	Other**	13(9.8)
Way of maternity ward to NICU transfer	Arm of staffs	116(87.9)
	Maternal arm	16(12.1)

NB:*Incubator and **Hypoglycemia, jaundice, congenital abnormality, and prematurity.

### Exploratory factor analysis

The scale demonstrated that very good sample adequacy for the Kaiser––Mayer––Olkin value was (KMO = 0.74). Bartlett’s test of sphericity that tests the null hypothesis that the original correlation matrix is an identity matrix was statistically significant (*χ*^2^ **=** 2903.9, DF = 276, P = 0.000). The extracted participation values of communalities ranged from 0.36 to 0.73. These all indicate that the data are suitable for EFA and can be grouped into a smaller number of factors.

### Factor extraction

The principal component analysis (PCA) extraction method was used and identified six retained factors with eigen values greater than one for further analysis. The six factors together explained 54.3% of the total variance in near-miss cases. The study revealed that the first factor explained 18.82% of the total variance. The second factor explained 8.23% of the total variance of the assessment scale. Likewise, Factor 3, Factor 4, Factor 5, and Factor 6 explained 8.09%, 7.12%, 6.36%, and 5.70% of the total variance of the scale, respectively (Table 2).

**Table 2.** Total variance explained for neonatal near-miss assessment scale in Amhara region public health hospitals, Northwest Ethiopia 2021 (n = 465)

Factors	Initial Eigenvalues			Extraction Sums of Squared Loadings		
	Total	% of Variance	Cumulative %	Total	% of Variance	Cumulative %
Cardio-respiratory	4.516	18.819	18.819	4.516	18.819	18.819
Sensory and drug	1.976	8.232	27.051	1.976	8.232	27.051
Neuro-renal	1.942	8.091	35.142	1.942	8.091	35.142
Hepatic	1.708	7.115	42.257	1.708	7.115	42.257
Lab-investigation	1.527	6.362	48.619	1.527	6.362	48.619
Pragmatic	1.367	5.696	54.315	1.367	5.696	54.315

### Factor rotation

The study indicates that the rotated matrix of neonatal nearmiss assessment-scale components was sufficient (> 0.30). The rotated component matrix revealed seven items loaded on factor 1 with factor loadings ranging from 0.525 to 0.816. Three items loaded on factor 2 with factor loadings were found between 0.573 and 0.700. Under the third factor, four items were loaded with factor loadings between 0.595 and 0.749. In the fourth factor, three items were loaded with loading factors ranging from 0.711 to 0.772. Three items were loaded on the fifth factor with factor loadings found between 0.625 and 0.852. Finally, four items were loaded on factor six with loading factors between 0.578 and 0.641 (Table 3).

**Table 3.** Rotated component matrix of exploratory factor analysis for neonatal near-miss assessment scale in Amhara region public health hospitals, Northwest Ethiopia 2021 (n = 465)

Rotated Component Matrix						
Number of items	Component					
	Cardio-respiratory	Sensory&drug	Neuro-renal	Hepatic	Laboratory	Pragmatic
Absence of regular breathing	.816					
Respiratory rate >70 bpm	.747					
Bradycardia <80 bpm	.743					
Positive pressure ventilation	.719					
Nasal-CPAP	.658					
Intubation for suctioning	.616					
APGAR score <7 at 5th minute	.525					
Use of corticosteroids		.700				
Inability to suck within 24 hrs		.693				
Use of vasoactive drugs		.573				
Neural tube defect			.749			
Recurrent seizure			.663			
Anuria greater than 6 hrs			.655			
Central cyanosis			.595			
Bilirubin level >10 mg/dl with in 24 hrs				.772		
Phototherapy within 24 hours				.768		
Jaundice in first 24 hours				.711		
Hgb < 10 g/dl,					.852	
WBC < 4000 cells/mm^3^					.769	
B/glucose level < 40 mg/dl in 24 hrs					.625	
First-week surgery						.641
Gestational age < 34 weeks						.632
Birth weight < 1750 grams						.579
Temperature 6–12 hrs <35°C						.578

### Factor labeling

In the pattern matrix, items within each factor were examined to label the factors. Seven items were loaded into factor 1 to identify neonatal near-miss cases and labeled as ‘cardio-respiratory domains.’ Three items loaded within a factor of 2 could identify near-miss cases and be labeled as ‘sensory and drug domains.’ Factor 3 included 3-items and was labeled as ‘neuro-renal domains.’ Three items were loaded under factor 4 and labeled as ‘hepatic domains.’ Factor 5 included three items and was labeled as ‘lab domains.’ Four items are loaded in factor 6 to identify susceptible near-miss cases and labeled as ‘pragmatic domains’ (Table 2).

### Internal consistency reliability analysis

NNMAS has good internal reliability with an overall Cronbach's alpha of 0.80. The study suggests that the scale is reliable. Because each of the items Cronbach's alpha and factor composite reliability ranges from 0.76 to 0.81 and 0.87 to 0.95, respectively. Items that have a higher correlation coefficient indicate a strong relationship between each item and the nature of the content intended to be measured. In the current study, 0.25 [34] was taken as the lower limit for item-total correlation (Table 4). The study also suggested that the mean, variance, and standard deviation were 1.53, 5.52, and 2.35, respectively, in the overall-scale statistics.

**Table 4.** Item total correlation, composite reliability (CR), the square root of the average variance extracted (**in bold**), and correlation b/n factors (**off-diagonal)** of NNMAS development and validation among live-born neonates in Amhara Region Public Health Hospitals, Northwest Ethiopia 2020 (n = 465)

Factor	N-o items	Item total correction range	CR	AVE	Factor Correlation Matrix					
					1	2	3	4	5	6
Factor 1	7	0.47–0.61	0.95	0.83	**0.91**					
Factor 2	3	0.42–0.47	0.87	0.81	.270	**0.90**				
Factor 3	4	0.35–0.43	0.90	0.80	.093	.057	**0.90**			
Factor 4	3	0.47–0.52	0.93	0.87	.163	.143	.059	**0.94**		
Factor 5	3	0.30–0.62	0.92	0.86	.147	.048	.057	.124	**0.93**	
Factor6	4	0.25–0.29	0.87	0.78	.092	.172	.012	.055	.045	**0.88**

### Convergent validity

It asks whether the measurement is related to variables to which it should be related if the items are valid. Convergent validity should be checked through factor loading, AVE, and CR value determination. Factor loading of all the factors was more than an acceptable level between 0.52 and 0.86 values after removing the low factor loading indicator. All of these factors have an acceptable level of AVE between 0.78 and 0.87. The study also suggested that the composite reliability of factors has more than acceptable levels between ranges of 0.87 to 0.95 (Table 3). Therefore, based on three conditions: factor loading, AVE, and CR for convergent validity were met.

### Discriminant validity

This was assured by using Fornell and Lacker in 1981 [37] by comparing the square root of each average variance extracted from the diagonal with the correlation coefficients (off-diagonal) for each factor in the relevant rows and columns [5]. As we have seen in the table, each of the square roots of average variance extractions is greater than each of the bottom columns and left-side row of factor correlation values. We concluded that discriminating validity can be accepted and supports the discriminant validity between factors (Table 4).

### Reliability and validity test discussions

This study reported on the psychometric evaluation of a Neonatal-Near Miss Scale for Ethiopia. It is a step forward in the direction of an innovative scale to improve the quality of care. We have developed NNMAS to assess cases of near-missing neonates and the results support the reliability and validity of the scale. NNMAS is easy to use, and it is intended to be implemented in low resource-limited settings and by all level health-care providers.

The development of the scale was associated with the application of exploratory factor analysis and further testing to establish reliable and valid measures (Internal Consistency, Composite Reliability, Convergent, and Discriminant validity) [12]. Reliable and valid NNMAS made a significant contribution to identifying ill-survival neonates early and predicting later development problems related to the life-threatening conditions to which those neonates were exposed. Thirty-one items were theoretically grouped under four domains for content validity. However, exploratory factor analysis grouped 23 items into 6 factors. Eight items: tachycardia, chest compression, vomiting, recurrent seizures, positive blood culture, cardiac arrest, blood exchange transfusion, and anticonvulsant drugs were deleted during factor extraction due to the low-cut value of the requirements.

The primary ‘cardio-respiratory domain (included seven items: Absence of regular breathing, respiratory rate > 70 bpm, Bradycardia < 80 bpm, positive pressure ventilation, nasal CPAP, Intubation for suctioning, and APGAR score less than 7 in the fifth minute)’ was near 19% of the total variance of the scale. But these items that were found in this domain could be found in more than two dimensions in other studies [22,39]. The reason could be due to the non-validation of the items in the previous study.

The second ‘sensory and drug-related domains (including three items: Inability to suck within 12 h, use of corticosteroid for hypoglycemia, and vasoactive drugs)’ was explained 8.3% of the total variance. However, in other studies, these items were found in two domains [22].

The third ‘neuro-renal domain (included four items: neural tube defects, recurrent seizures, anuria greater than 6 h, and central cyanosis)’ was 8.1% of the total variance in the study. However, items in this dimension were found under different domains in other studies [40]. It was not validated like the above studies.

The fourth dimension created through the factor analysis was ‘hepatic domain’ (included three items: Serum bilirubin level > 10 mg/dl within 24 h, Jaundice in first 24 h, and Phototherapy within 24 h) was 7.1% of the total variance. The constructs were found in different dimensions in other studies [39–41].

The fifth dimension ‘lab-investigation domain’ (included three items: Hgb <10 g/dl, WBC < 4000 cells/mm^3^, and Blood glucose level < 40 mg/dl in 24 h) was 6.4% the variance, and two items were (positive blood culture and bilirubin level) removed. This dimension was in line with a study conducted in Ghana but not at the item levels [39]. It could be due to a lack of validation of the scale in this study.

The sixth dimension was ‘pragmatic domain’ (included four items: first-week surgery, a gestational age of less than 1750 g, a birth weight of less than 34 weeks, and an auxiliary temperature 6–12 h less than 35°C). Similarly, factor analysis made four items under this domain except for the exchange of APGAR score less than 7 at the 5th minute by first-week surgery). This was the last factor that explained 5.7% of the total variance in the study. In this study, all the factors discriminated through factor analysis supported Neonatal Near-miss Assessment Scale as a multidimensional factor.

NNMAS was found to have good internal reliability for the total scale. Internal consistency of NNMAS was found at a Cronbach's alpha coefficient of **0.80**. The study also suggested that the composite reliability of the factors ranged from 0.87 to 0.95 in primary to the end of dimensions.

The three conditions for convergent validity determination were factor loading; AVE and composite reliability met the validation process. In this study, the square roots of Average Variance Extractions (AVE) were greater than each of the bottom columns and left-side row of factor correlation values. Finally, the study revealed that discrimina validity could be accepted and supports the discriminant validity between factors.

## Strength and limitations

The study included a large sample of neonates strengthening trustworthiness. Neonates are considered representative of all neonates in neonatal wards in Ethiopia with randomly selected maternity and neonatal wards in mind. No significant difference was found in the medical records or care data of the neonates. In this study, we have used the principal component analysis method for factor rotation and determination. However, the method might violate the multicollinearity assumption. The cross-sectional design of the study, however, facilitated the possibility to validate NNMAS for test–retest reliability.

## Conclusion

The neonatal near-miss assessment scale was found to be valid and reliable in the present context. The scale can be used to identify near-miss neonates in Ethiopia.

## Clinical implications

This study will be a benchmark in Ethiopia and possibly the validated neonatal near-miss scale will help to improve the quality of care. In addition, the results of this study will contribute to the body of knowledge on validation of neonatal near-miss scales and the neonatal near-miss concept in general. Finally, the scale can be used to inform policymakers and programmers on how best to apply scarce resources to improve the quality of care and reduce neonatal mortality. More similar studies are welcome to build evidence on the Neonatal-Near Miss concept and scale validation. Our results generalize the fact that the scale is suitable and can be used in low-resource settings. Implementing a reliable and valid NNMAS for ill-survival neonates identification has made a significant contribution to knowledge generation and quality care improvements.

## Paper context

In Ethiopia, the neonatal death estimate is high. The concept of a neonatal near miss is used to identify why neonates who nearly died survived from a life-threatening complication during the first 28 days of life. This paper adds a valid and reliable neonatal near-miss assessment scale for the Ethiopian context. This study provides a validated and reliable scale that can be used to identify neonatal near-miss cases in Ethiopia.
